# Childhood tuberculosis and its treatment outcomes in Addis Ababa: a 5-years retrospective study

**DOI:** 10.1186/1471-2431-14-61

**Published:** 2014-03-03

**Authors:** Dereje Hailu, Woldaregay Erku Abegaz, Mulugeta Belay

**Affiliations:** 1 Addis Ababa Health and Research Laboratory, P.O.Box 30738, Addis Ababa, Ethiopia; 2 Aklilu Lemma Institute of Pathobiology, Addis Ababa University, P.O.Box 1176, Addis Ababa, Ethiopia

**Keywords:** TB, Children, Treatment outcomes, Ethiopia

## Abstract

**Background:**

Tuberculosis (TB) remains a significant public health problem leading to high morbidity and mortality both in adults and children. Reports on childhood TB and its treatment outcome are limited. In this retrospective study, we analyzed the epidemiology and treatment outcomes of TB among children in Addis Ababa.

**Methods:**

Children registered for TB treatment over 5 years (2007 to 2011) were included in the analysis. Demographic and clinical data including treatment outcomes were extracted from TB unit registers of 23 health centers in Addis Ababa. Multivariate logistic regression was used to identify predictors of poor treatment outcomes.

**Results:**

Among 41,254 TB patients registered for treatment at the 23 health centers, 2708 (6.6%) were children. Among children with TB, the proportions of smear positive PTB, smear negative PTB and EPTB were 9.6%, 43.0% and 47.4%, respectively. Treatment outcomes were documented for 95.2% of children of whom 85.5% were successfully treated while rates of mortality and defaulting from treatment were 3.3% and 3.8%, respectively. The proportion of children with TB tested for HIV reached 88.3% during the final year of the study period compared to only 3.9% at the beginning of the study period. Mortality was significantly higher among under-five children (p < 0.001) and those with HIV co-infection (p < 0.001). On multivariate logistic regression, children 5–9 years [AOR = 2.50 (95% CI 1.67-3.74)] and 10–14 years [AOR = 2.70 (95% CI 1.86-3.91)] had a significantly higher successful treatment outcomes. On the other hand, smear positive PTB [AOR = 0.44 (95% CI 0.27-0.73), HIV co-infection (AOR = 0.49(95% CI 0.30-0.80)] and unknown HIV sero-status [AOR = 0.60 (95% CI 0.42-0.86)] were predictors of poor treatment outcomes.

**Conclusion:**

The proportion of childhood TB in this study is lower than the national estimate. The overall treatment success rate has met the WHO target. Nonetheless, younger children (< 5 years), children with smear positive PTB and those with HIV co-infection need special attention to reduce poor treatment outcomes among children in the study area.

## Background

Tuberculosis (TB) is one of the major public health problems worldwide. In 2012 alone, there were 8.6 million new cases and 1.3 million deaths globally [1]. Although the true burden of childhood TB is not well known, it is one of the 10 major causes of childhood mortality with estimated annual deaths of 74,000 [1] to 130,000 [2]. Besides, it is estimated that about 6% of new cases of TB occur in children [1,3]; however, this proportion varies with the prevalence of TB in adults ranging from ~5% in low-burden countries to 20-40% in high-burden countries [2]. More than 75% of children with TB are from the 22 high-burden countries [2,4]. In Ethiopia, one of the 22 high TB burden countries, TB is the second leading cause of death [5]. It is estimated that children contribute to 16.1% of the national TB burden [3]. In an effort to control the disease, the country adopted the WHO DOTS strategy as a standardized TB prevention and control programme in 1992.

Childhood TB is a marker of recent transmission in a population; moreover, children are the primary victims of a poor TB control programme [3]. The highest priority, however, has been given to infectious TB cases (mostly of adults) and the management and prevention of TB among children is relatively neglected despite the fact that TB is a cause of significant childhood mortality and morbidity [1,3]. In TB endemic countries, delayed diagnosis and high case density are major factors contributing to continued transmission [6]. In addition, epidemiological data on childhood TB is limited [4] mainly because of absence of surveillance data as well as poor ascertainment of cases [6].

In low income countries, children with respiratory infections present with multiple infectious diseases including TB [7] complicating diagnosis and proper treatment. Because the routine diagnostic test for TB is smear microscopy, correct diagnosis of TB is difficult among the majority of children especially the young since either they do not produce sputum or have paucibacillary sputum. Thus, diagnosis in these patients heavily depends on clinical history (suggestive symptoms, poor response to a course of antibiotics, contact to known PTB patients) and physical examination including growth assessment and chest x-ray.

Surveillance data on childhood TB is important to define its epidemiology and identify predictors of poor treatment outcomes. WHO recommends that children with TB should be treated and notified through the national TB control programme [4]. However, like any other resource-poor countries, such reports in Ethiopia are mainly limited to adults with infectious TB patients. In Ethiopia, apart from studies in rural areas [8,9], the contribution of childhood TB as well as its treatment outcomes is not well documented. This study, therefore, investigated the treatment outcomes of TB and its predictors among children in an urban setting.

## Methods

### Study area

This study was conducted in Addis Ababa which is home to about 2.7 million people [10]. Administratively, the city is divided into 10 sub-cities and 116 *Woredas* which are the lowest administrative units. The public health institutions in the city include 10 hospitals & 26 health centers. In addition, there are 36 hospitals and over 400 clinics run by the private sector. TB treatment was limited to public health facilities mainly health centers until 2006 when some selected private health facilities were included as pilot sites [11]. The Public-Private Mix program has been progressively expanded since then. By 2011, 9.5% of TB patients were detected at the private health facilities nationwide [11]. The majority of TB patients diagnosed at hospitals (both government and private owned) and private clinics were mainly referred to the nearest health centers for treatment. Therefore, this study included 23 of the total 26 health centers which were providing DOTS service during data collection; the remaining 3 health centers were excluded since they started the service recently (< 1 year).

### TB diagnosis and treatment in children

According to the Ethiopian National TB and Leprosy Control Program (NTLCP) [5], patients having cough lasting for at least 2 weeks should have smear microscopic examination of their sputum. Clinical history, chest x-ray, HIV testing and histopathology are used to diagnosis smear negative pulmonary TB (PTB) and extra pulmonary TB (EPTB). Among PTB suspects, clinical diagnosis is made if two of these features are present: positive contact history, suggestive physical signs, and suggestive chest x-ray findings. Besides, chest x-ray with miliary feature, bacteriological evidence (smear or culture positive) or histopathological evidence alone could be taken as an evidence to diagnose TB. Treatment of new TB patients consists of a 2-month intensive phase followed by a 4-month continuation phase. During the intensive phase, 4 drugs (Rifampicin, Isoniazid, Pyrazinamide and Ethambutol) are taken daily under the supervision of a health worker. In the continuation phase, two drugs (Rifampicin and Isoniazid) are taken every day and in this phase parents/caregivers are in charge of supervising adherence to treatment.

### Study design and data collection

A retrospective data analysis was done on the treatment outcomes of children (<15 years) with TB who were registered for treatment from January 2007 until December 2011 at health centers in Addis Ababa. Data on demographics (age and sex), types of TB, smear result (baseline and follow-up for smear positive PTB patients), categories of TB, HIV sero-status and treatment outcomes were extracted from TB unit registers of each health center. Standard definitions of the Ethiopian NTLCP guideline [5] for categories, types of TB and treatment outcomes were used. Data were extracted from TB unit registers of each health center by a trained nurse; and one of the authors supervised data collection.

### Operational definitions

#### Treatment success

The sum of patients who were declared “cured” and “treatment completed”.

#### Poor treatment outcome

Includes patients who were documented as “died”, “defaulted”, “treatment failures” and “transferred out”.

### Data analysis

The data extracted from TB unit registers were checked for completeness and accuracy. Data were entered into excel and exported to SPSS version 20 for analysis. Chi-square test was used for categorical variables to evaluate associations between dependent and independent variables. Since age was not normally distributed, Mann–Whitney test was used to analyze the association between age and smear positivity among PTB patients. Multivariate logistic regression was performed to identify predictors of treatment outcomes. The association of predictor variables with the dependent variable was described using 95% confidence interval (CI) and adjusted odds ratio (aOR). A p-value < 0.05 was considered statistically significant.

### Ethical consideration

Ethical clearance was obtained from the Institutional Review Board of Aklilu Lemma Institute of Pathobiology, Addis Ababa University and Addis Ababa City Administration Health Bureau. To maintain confidentiality, names or other identifiers of study participants were not included.

## Results

**Figure 1:**
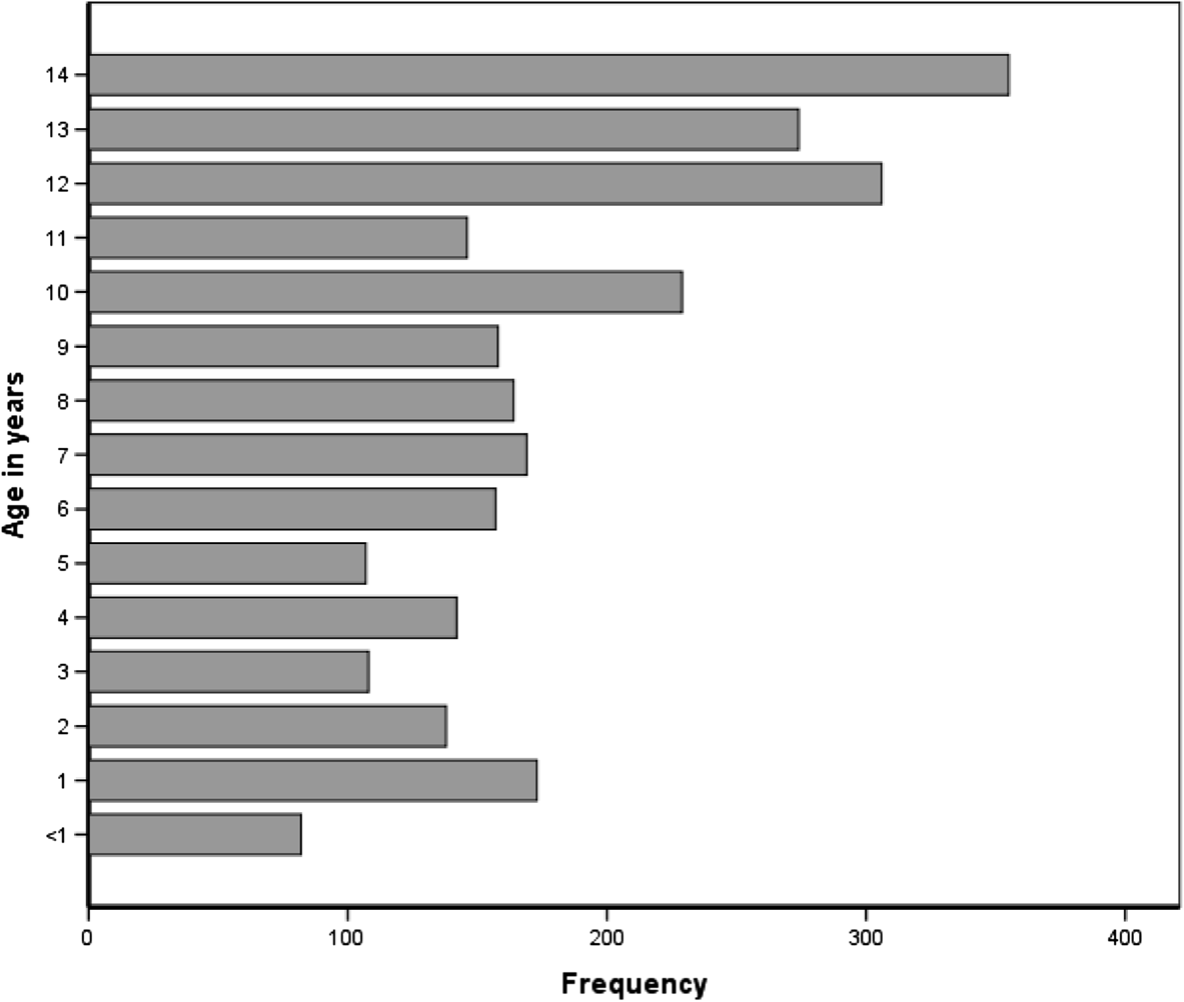
Age distribution of children with TB in Addis Ababa, 2007–2011.

Over 5 years, a total of 41,254 TB patients were registered for treatment at the selected health centers, of whom 2708 (6.6%) were children. Overall, childhood TB contributed to 2.4% of smear positive PTB, 7.6% of smear negative PTB and 8.4% of EPTB patients. Among children with TB, the median age was 9 (IQR 5–12) years, and 52.7% of them were females. Nearly half of children with TB were 10 years or older whereas those younger than 5 years accounted for 23.7% (Figure 1). The majority (88.1%) of children were registered as new TB patients whereas 6.7% were transferred in from other health facilities. Twenty-three (0.9%) children with TB were registered as retreatment cases. EPTB accounted for nearly half (47.4%) of childhood TB (Table 1). Among children <5 years, the proportion of EPTB is slightly lower (44.8%) compared to older children.

**Table 1:** Treatment outcomes of children with TB in Addis Ababa, 2007-2011

**Characteristics**	**Successfully treated**		**Poor treatment outcome**				
	**Cured (n = 169)**	**Completed treatment (n = 2024)**	**Died (n = 83)**	**Failed (n = 6)**	**Defaulted (n = 99)**	**Transfer out (n = 184)**	**p-value**
**Age**							
0–4	4(0.7)	461(77.1)	36(6.0)	1(0.2)	37(6.2)	59(9.9)	<0.001
5–9	22(3.0)	626(86.0)	22(3.0)	0(0)	21(2.9)	37(5.1)	
10-14	143(11.4)	947(75.7)	26(2.1)	5(0.4)	41(3.3)	89(7.1)	
**Sex**							
Male	59(4.9)	989(81.5)	37(3.0)	2(0.2)	45(3.7)	82(6.7)	0.02
Female	109(8.1)	1049(77.0)	47(3.4)	2(0.1)	53(3.9)	103(7.6)	
**Type of TB†**							
PTB + ^*^	167(67.1)	37(14.9)	10(4.0)	4(1.6)	13(5.2)	18(7.2)	<0.001
PTB-^**†**^	NA*	944(85.4)	35(3.2)	NA*	47(4.3)	79(7.1)	
EPTB**‡**	NA*	1054(86.6)	37(3.0)	NA*	38(3.1)	88(7.2)	
**Category**							
New	157(7.0)	1782(78.9)	65(2.9)	3(0.1)	90(4.0)	161(7.1)	<0.001
Relapse	7(41.2)	9(52.9)	0(0)	0(0)	0(0)	1(5.9)	
Treatment failure	0(0)	0(0)	0(0)	1(100)	0(0)	0(0)	
Defaulter	0(0)	3(100)	0(0)	0(0)	0(0)	0(0)	
Others	0(0)	94(83.9)	8(7.1)	0(0)	2(1.8)	8(7.1)	
**HIV**							
Negative	72(7.9)	729(80.2)	18(2.0)	2(0.2)	26(2.9)	62(6.8)	<0.001
Positive	17(5.2)	260(79.8)	22(6.7)	2(0.6)	8(2.5)	17(5.2)	
Unknown	79(5.9)	1044(78.2)	43(3.2)	0(0)	64(4.8)	105(7.9)	

*NA = not applicable; ^*^PTB + = Smear positive PTB; ^**†**^PTB- = Smear negative PTB; **‡**EPTB = Extra pulmonary TB.

†chi-square test was done after combining “cured” and “treatment completed”.

Among children diagnosed with PTB, only 18.2% were smear positive. Smear positivity was significantly associated with HIV infection, age and sex of children. A significantly higher proportion of HIV positives were diagnosed with smear negative PTB compared to HIV negatives (p < 0.001). The majority (80%) of smear positive PTB patients were 10 years or older (median 13 yrs, IQR 10–14 yrs) compared to children with smear negative PTB (median 8 yrs, IQR 4–12 yrs) (Mann–Whitney, p < 0.001). A significantly higher proportion of females than males had smear positive PTB (p < 0.001) (Table 2).

**Table 2:** Factors associated with types of TB among children with TB in Addis Ababa

**Characteristics**	**PTB+**	**PTB-**	**EPTB**	**p-value**
**Sex**				
Male	91(7.1)	576(45.1)	611(47.8)	
Female	168(11.8)	486(41.2)	667(46.9)	<0.001
**Age**				
0-4	11(1.7)	342(53.8)	286(44.8)	
5-9	40(5.3)	359(47.7)	354(47.0)	<0.001
10-15	208(15.9)	461(35.3)	638(48.8)	
**HIV infection**				
Negative	105(11.1)	377(39.9)	463(49.0)	
Positive	28(8.1)	215(62.1)	103(29.8)	<0.001
Unknown	125(8.9)	568(40.6)	707(50.5)	
**Category**				
New	236(10.0)	1023(43.2)	1109(46.8)	0.14
Others	23(6.9)	139(42.0)	169(51.1)	

Of the total 259 children with smear positive PTB, sputum smear microscopy was done for 207 (79.9%) at the end of the second month of treatment and 6 (2.3%) were documented as smear positive. At the end of the 5^th^ month, 3/189 (1.2%) were smear positive and one additional patient who was smear negative at the 5^th^ month became smear positive on completion of treatment, making the number of children who failed to respond to first line anti-TB drugs 4/259 (1.5%). Overall, 65.3% of the smear positive PTB cases were reported to be cured based on follow-up smear results.

The proportion of children with TB who were tested for HIV progressively increased over the years. In 2007, only 3.9% of the children with TB were tested for HIV; however, HIV testing has increased to 62.7% and 88.3% in 2009 and 2011, respectively. Overall, HIV status was known for 47.9% of children and among these, 26.8% were co-infected with the virus. Children younger than 10 years had a significantly higher proportion of HIV co-infection (33.8%) compared to older children (19.8%) (p < 0.001).

**Figure 2:**
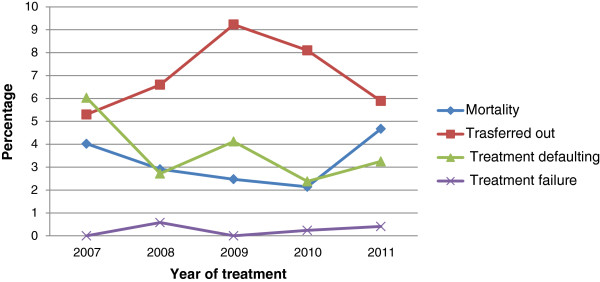
Trends of mortality, treatment defaulting, treatment failure and transfer out among children with TB in Addis Ababa, 2007–2011.

Among the 2708 children registered for treatment, 131 (4.9%) had no documented treatment outcomes. Of the 2579 children with documented treatment outcomes, 85.5% were successfully treated (Table 1). Although the overall treatment success was high, there was a significant variation across the health centers, from as low as 76% to as high as 96% (p = 0.002). Children younger than 5 years had a treatment success of 78.1%, significantly less than the treatment success among older children (> = 87.3%) (p < 0.001). Similarly, treatment success varied with clinical forms of TB, patients with smear positive PTB having a lower (81.9%) treatment success compared to patients with either EPTB (86.6%) or smear negative PTB patients (85.4%) (p = 0.15). On the otherhand, treatment outcomes were similar among males (86.3%) and females (85.0%) (p = 0.35). Similarly, there was no significant difference in the overall treatment success rate over the five years (84.4% to 86.7%).Eighty-four (3.3%) and 99 (3.8%) of children registered for treament were documented as dead and defaulted, respectively. The default rate has dropped from 6.2% in 2006 to 3.8% in 2011, whereas mortality decreased from 4% in 2006 to 2% in 2010; however, the reduction in poor outcomes over the years is not significantly different (p for trend = 0.17) (Figure 2). Mortality was significantly higher among HIV infected (6.7%) compared to HIV negative children (2%) (p < 0.001). Moreover, those younger than 5 years had a significantly higher mortality (6%) and defaulting rate (6%) compared to older children (mortality and defaulting rates of 2.4% and 3.1%, respectively) (p < 0.001).

On multivariate logistic regression, age group 5–9 years [AOR = 2.50 (95% CI 1.67-3.74)] and 10–14 years [AOR = 2.70 (95% CI 1.86-3.91)] were independently associated with successful treatment outcomes. On the other hand, patients with smear positive PTB [AOR = 0.44 (95% CI 0.27-0.73), those co-infected with HIV virus (AOR = 0.49(95% CI 0.30-0.80)] and those with unknown sero-status [AOR = 0.60 (95% CI 0.42-0.86)] had significantly lower treatment success rates (Table 3).

**Table 3:** Predictors of successful treatment outcomes among children with TB, Addis Ababa

**Characteristics**	Successfully treated*	**Not successfully treated**	**AOR (95% CI)**
	**Frequency (%)**	**Frequency (%)**	
**Age**			
0-4	466(86.6)	72(13.4)	1.00
5-9	648(93.8)	43(6.2)	2.50(1.67-3.74)
10-14	1093(94.0)	70(6.0)	2.70(1.86-3.91)
**Type of TB**			
PTB-	944(92.0)	82(8.0)	1.00
PTB+	204(88.3)	27(11.7)	0.44(0.27-0.73)
EPTB	1054(93.4)	75(6.6)	1.09(0.78-1.52)
**HIV infection**			
Negative	801(94.6)	46(5.4)	1.00
Positive	277(89.6)	32(10.4)	0.49(0.30-0.80)
Unknown	1123(91.3)	107(8.7)	0.60(0.42-0.86)

*Transferred out patients were excluded from the Logistic Regression Model since their treatment outcome is unknown. The model was adjusted for sex.

## Discussion

This study investigated the treatment outcomes of children registered for TB treatment at health centers of Addis Ababa. Children contributed to 6.6% of the total TB patients registered for treatment which is in agreement with a previous study in 3 health centers of Addis Ababa [12] but much lower than the proportion reported in Southern Ethiopia [8,9] and the national estimate (16.1%) [3]. Delayed diagnosis among adult TB patients has been reported to be a serious problem both in rural [13,14] and urban [15] Ethiopia, and therefore, it is likely that continued transmission with high childhood TB exist in the country. Nonetheless, significant variations between communities are common without clear explanations [16]. Misdiagnosis of childhood TB especially among the young is a common problem in TB endemic countries [6] which might have contributed to a relatively low proportion of childhood TB in our study. This is substantiated by the fact that under-five children represented only a small proportion of children with TB in our study although the young are said to be more vulnerable to disease progression [3].

The risk of progression to active TB is greatest among young children especially those below 2 years [17] and under-five children constituted the majority of childhood TB in previous studies in Africa [18,19] and Thailand [20]. However, in our study, older children (10–14 years) represented nearly half (48.4%) of the registered cases. Although not evident from the current study, it is probable that TB among young children might have been missed.

The majority (81.7%) of children with PTB were smear negative which is in agreement with previous reports [8,21]. This is mainly because most children present with primary rather than secondary TB (with cavitory lesions) and therefore, are likely to have low bacillary load. Moreover, young children do not produce sputum for smear microscopy and are diagnosed based on clinical and chest x-ray evidences. The proportion of children with EPTB (47.4%) in this study is comparable to a recent report (40.0%) both in adults and children from Addis Ababa [12]. However, the proportion of EPTB in our study is much higher compared to two recent reports from Southern Ethiopia (24.8%) [6] and (31.1%) [7] and Malawi [18]. The relatively low TB burden together with a high proportion of EPTB among children in our study suggests that further epidemiological study on childhood TB is important.

Regarding treatment outcomes, the overall treatment success rate of 85.5% has met the WHO target. The treatment success rate in this study is higher compared to previous reports both from Ethiopia [8,9] and elsewhere in Africa [19,21,22]. This difference might be related to differences in setting, disease presentation as well as prevalence of HIV infection. Besides, there might be differences in the level of adherence which is mainly dependent on the parents’ level of supervision and administration of medication especially among young children.

The mortality rate (3.3%) in this study is comparable to the mortality rate (3.7%) reported for both adults and children in Addis Ababa [12] but is lower compared to previous reports from other parts of Ethiopia (5.8% and 5.3%) [8,9], Botswana (10.5%) [22], Tanzania (10.9%) [21] and Malawi (17%) [19]. In this study, the majority of children were tested for HIV in the later years of the study period, which might have partly contributed to better management of TB and other HIV related infections thereby reducing mortality. Alternatively, the lack of data on the treatment outcomes of transferred out and defaulted children among whom mortality is expected to be higher might have resulted in underestimating the mortality rate.

A number of factors associated with treatment outcomes have been reported. In this study, smear positive PTB was found to be associated with poor treatment outcome as previously reported [23]. In contrast to our finding, however, some studies [8,19] reported that having smear positive PTB is associated with favorable treatment outcomes. However, it might be reasonable to assume that children with smear positive PTB to have advanced disease at presentation, resulting in poor treatment outcomes.

The treatment success rate among children younger than 5 years was low with a significantly higher mortality and defaulting rates. In agreement with this finding, a study in Malawi [19] reported a decline in the death and defaulting rates with advanced age. Younger children especially those under two years are at a greater risk of death from infectious diseases including TB because of immature immune systems. Moreover, disseminated TB and TB meningitis, both associated with high mortality, are more common among young children [2,3,24]. Diagnosis of TB in younger children remains a challenge and most end up with anti-TB treatment without confirmation. This would lead to delay in the diagnosis and treatment of other serious illnesses especially HIV-related opportunistic infections resulting in increased mortality.

The proportion of children tested for HIV progressively increased over the years, from 3.9% in 2007 to 88.1% by the end of the study period. Introduction of provider-initiated counseling and testing has probably resulted in a recent increase in the proportion of those tested for the virus. Among tested, TB-HIV co-infection was found to be 26.8%. However, the co-infection rate considerably varied with age, older children having the least co-infection rate. Knowledge of HIV sero-status is essential for better management of TB as well as HIV/AIDS. In this study, co-infection with HIV was found to be an independent predictor of unfavorable treatment outcome with significant mortality and this is in agreement with previous reports elsewhere [21,22,25]. HIV co-infection is commonly associated with multiple infections complicating diagnosis as well as treatment [7] and hence leading to greater morbidity and mortality.

This study is not without limitations and therefore, the findings of this study should be interpreted in view of the following limitations. First, socioeconomic data including family income and educational status of parents which might influence treatment outcomes were not documented and therefore, the role of such variables was not investigated. In addition, not all health facilities providing DOTS were included in this study, which might limit the generalizability of our findings to all children with TB registered for treatment in Addis Ababa.

## Conclusions

The proportion of childhood TB in the study area is lower than expected given the high prevalence of smear positive TB in adults as well as the HIV epidemic. Investigating the diagnostic procedures at health facilities is important to have an insight on the burden of childhood TB. The overall treatment success rate in the current study has met the WHO target of 85% and is higher compared to previous reports from Ethiopia and Africa. However, the outcomes of treatment varied with age, HIV status and clinical forms of TB. Young children, those co-infected with HIV and those with smear positive PTB need special attention to reduce unfavorable treatment outcomes among children.

## Competing interests

The authors declare that they have no competing interests.

## Authors’ contributions

DH: Collected the data and involved in the data analysis; MB: Designed the study, analyzed & interpret the data and drafted the manuscript; WEA: Involved in the design, interpretation and critical revision of the manuscript. All approved the final version of the manuscript.

## Pre-publication history

The pre-publication history for this paper can be accessed here:

http://www.biomedcentral.com/1471-2431/14/61/prepub
